# Supplementary material for: Mapping the Intersection of Research and Policy in Centers for Medicare National Coverage Decision Memos
Source: ArXiv. 2025 May 1:arXiv:2505.00854v1. Preprint. [Version 1] (PMC12306809)
Supplement: 1 [file NIHPP2505.00854V1-supplement-1.pdf]

Supplement

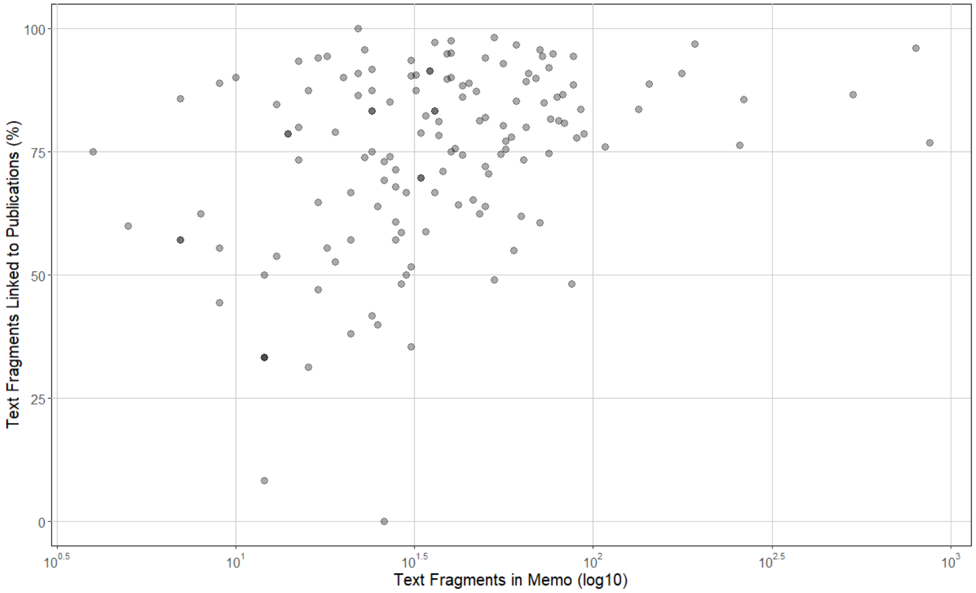

Supplementary Figure 1. Relationship between amount of memo text and percentage of memo text linked to a publication. Text fragments are free text extracted from memo reference sections and are checked to see if they match a publication in PubMed (see Methods). The x-axis is on $\log_{10}$ scale. Points represent memos (N = 146).

Supplementary Table 1

Percentage and Difference of Shares of Funders Supporting Memo Articles

Funder	Awards (#)	Awards (%)	N	Median Difference	p-value
Agency for Healthcare Research & Quality	38	1.39			
Arthritis Research UK	2	0.07			
Agency for Toxic Substances & Disease Registry	2	0.07			
Austrian Science Fund FWF	1	0.04			
Bureau of Health Professions	1	0.04			
Brazilian National Council for Scientific and Technological Development	2	0.07			
British Heart Foundation	10	0.36			
Canadian Institutes of Health Research	19	0.69			
Cancer Research UK	5	0.18			
Chief Scientist Office	12	0.44			
CLC	4	0.15			
DS	1	0.04			
Fundação de Apoio à Pesquisa do RN	1	0.04			
U.S. Food & Drug Administration	6	0.22			
FIC	2	0.07			
The São Paulo Research Foundation	1	0.04			
U.S. Centers for Disease Control & Prevention	2	0.07			
Intramural NIH	20	0.73			
Medical Research Council	51	1.86			
Ministry of Health and Welfare	1	0.04			
Ministry of Science and Technology, Taiwan	3	0.11			
Multiple Sclerosis Society	2	0.07			
NCATS	200	7.29	33	6 (4.5, 9.6)	3E-09
NCCAM	7	0.26			
NCCDPHP	2	0.07			
NCCIH	2	0.07			

NCHHSTP	2	0.07				
NCI	566	20.64	35	11	(6.8, 14.3)	7E-08
NCMHD	3	0.11				
NEI	28	1.02	12	0	(-1.2, 2.2)	1E+00
NHGRI	1	0.04				
NHLBI	552	20.13	34	14	(10.1, 16.4)	2E-09
NIA	335	12.22	30	7	(5.9, 13.7)	4E-09
NIAID	79	2.88	16	-6	(-7.1, -4)	6E-03
NIAMS	58	2.12	23	1	(0.2, 2.1)	3E-02
NIBIB	13	0.47	10	0	(-0.5, 0.4)	6E-01
NICHD	23	0.84	11	-3	(-3.2, -2.3)	1E-03
NIDA	45	1.64	14	-1	(-1.5, 0.4)	3E-01
NIDCD	4	0.15				
NIDCR	7	0.26	6	0	(-0.5, 1.1)	6E-01
NIDDK	234	8.53	33	0	(-0.8, 8)	2E-01
NIEHS	13	0.47	9	0	(-0.9, 0.8)	9E-01
NIGMS	36	1.31	19	-8	(-8.2, -5.4)	4E-06
NIH	2	0.07				
NIMH	89	3.25	24	-2	(-2.3, -0.3)	2E-02
NIMHD	5	0.18				
NINDS	108	3.94	27	-2	(-2.6, 0.3)	1E-01
NINR	8	0.29	8	0	(0, 2.1)	3E-02
NIOSH	1	0.04				
NLM	4	0.15				
Office of Chief of Public Health Practice	1	0.04				
PHS	86	3.14				
Coord. for the Improvement of Higher Education Personnel Nat. Postdoc. Program	1	0.04				
SingHealth Foundation Research Grant	1	0.04				

Telethon	1	0.04
UK Department of Health	17	0.62
U.S. Dept. of Veterans Affairs	2	0.07
Wellcome Trust	20	0.73

Notes. The percent of awards is calculated as the award number over the total number of awards (N = 2,742). Median difference is calculated using equation 1 with bracketed numbers indicating the bounds of a 95% confidence interval. Positive values indicate the IC is overrepresented in the memo data while negative values indicate it is underrepresented. P-values are estimated from a Wilcoxon signed rank test. Acronyms in alphabetical order: National Center for HIV, Viral Hepatitis, STD, and Tuberculosis Prevention (NCHHSTP), National Institute for Occupational Safety and Health (NIOSH), Public Health Services (PHS), all other acronyms are NIH Institutes, Centers, or Offices that can be found at (NIH, 2022).

References

NIH. (2022). *Deciphering NIH Application/Grant Numbers*. National Institutes of Health. https://www.era.nih.gov/files/Deciphering_NIH_Application.pdf

Supplemental Table 2

Differences in article preferences between CMS policy staff and scientists by memo.

Memo Title	# Memo Articles w/ RCRs	Median ΔRCR		# Memo Articles w/ Year	Median $\Delta Year$		KLD_F	KLD_{RO}
Decision Memo for Ambulatory Blood Pressure Monitoring (ABPM) (CAG-00067R2)	25	3.3	(0.9, 30)	25	-3	(-4, -1)	0.5	0.6
Decision Memo for Arthroscopy for the Osteoarthritic Knee (CAG- 00167N)	30	-0.8	(-1.4, 0.9)	30	1	(-4, 3)	0.1	0.1
Decision Memo for Autologous Blood-Derived Products for Chronic Non-Healing Wounds (CAG-00190N)	41	-0.7	(-1.1, -0.1)	41	-2	(-3, 1)	0.1	0.1
Decision Memo for Autologous Blood-Derived Products for Chronic Non-Healing Wounds (CAG-00190R3)	64	0	(-0.4, 0.8)	64	-1	(-3, 1.5)	0.0	0.0
Decision Memo for Autologous Stem Cell Transplantation (AuSCT) for Multiple Myeloma (CAG-00011N)	20	0.1	(-0.3, 2.2)	20	-2	(-3.5, -1.5)	0.0	0.2
Decision Memo for Bariatric Surgery for the Treatment of Morbid Obesity - Facility Certification Requirement (CAG-00250R3)	28	-0.4	(-0.8, 0.2)	28	-2	(-3, 0)	0.5	0.7
Decision Memo for Beta Amyloid Positron Emission Tomography in Dementia and Neurodegenerative Disease (CAG-00431N)	85	4.8	(3.6, 7.5)	85	-4	(-4, -3)	1.7	0.4
Decision Memo for Blood Brain Barrier Disruption (BBBD) Chemotherapy (CAG-00333N)	39	0	(-0.7, 1.3)	40	6	(4, 8.5)	0.3	0.9
Decision Memo for Carotid Artery Stenting (CAG-00085R)	23	9.6	(5, 23.1)	23	-1	(-3, -1)	0.3	0.2
Decision Memo for Chimeric Antigen Receptor (CAR) T-cell Therapy for Cancers (CAG-00451N)	30	13	(6.4, 23.2)	30	-1	(-2, 2)	0.9	0.7
Decision Memo for Collagen Meniscus Implant (CAG-00414N)	48	0.6	(-0.1, 1.4)	51	-1	(-3, 2)	0.0	0.0

Decision Memo for Computed Tomographic Angiography (CAG- 00385N)	44	1	(-0.6, 3.7)	45	-4	(-4, -4)	0.1	0.0
Decision Memo for Continuous Positive Airway Pressure (CPAP) Therapy for Obstructive Sleep Apnea (OSA) (CAG-00093R)	24	-1	(-1.5, 0.2)	24	-1	(-2, 1)	0.5	0.4
Decision Memo for Continuous Positive Airway Pressure (CPAP) Therapy for Obstructive Sleep Apnea (OSA) (CAG-00093R2)	62	0.6	(0.1, 1)	62	-1	(-2.5, 1)	0.1	0.1
Decision Memo for Electrical Bioimpedance for Cardiac Output Monitoring (CAG-00001R)	90	-1.2	(-1.4, -0.9)	91	0	(-1, 1)	0.1	0.1
Decision Memo for Electrodiagnostic Sensory Nerve Conduction Threshold (CAG-00106R)	36	-1.5	(-1.8, -0.9)	36	0	(-4.5, 1)	0.0	0.0
Decision Memo for Electrostimulation for Wounds (CAG-00068N)	34	-0.3	(-1, 0.4)	40	2.5	(1, 6)	0.1	0.0
Decision Memo for Erythropoiesis Stimulating Agents (ESAs) for non-renal disease indications (CAG-00383N)	498	-0.6	(-0.8, -0.4)	503	2	(1, 3)	0.9	0.6
Decision Memo for External Counterpulsation (ECP) Therapy (CAG- 00002R2)	44	-2	(-2.2, -1.7)	46	-3	(-5, -2.5)	0.0	0.0
Decision Memo for Extracorporeal Photopheresis (CAG-00324R)	34	0.4	(-0.2, 1.2)	35	-1	(-3, 2)	0.2	0.1
Decision Memo for Extracorporeal Photopheresis (ECP) (CAG- 00324R2)	37	1	(-0.1, 2.2)	39	6	(1, 7)	0.2	0.1
Decision Memo for Ferrlecit®: Intravenous Iron Therapy (CAG- 00046N)	24	-0.1	(-1.5, 1.1)	24	-2	(-4, -1.5)	0.0	0.0
Decision Memo for Gender Dysphoria and Gender Reassignment Surgery (CAG-00446N)	301	-0.1	(-0.3, 0.3)	335	0	(-1, 1)	0.3	0.2
Decision Memo for Heartsbreath Test for Heart Transplant Rejection (CAG-00394N)	36	-0.3	(-0.6, 0.4)	36	5.5	(3, 8)	0.2	0.6
Decision Memo for Implantable Cardioverter Defibrillators (CAG- 00157R4)	43	3.8	(1.5, 10.1)	43	4	(1, 7)	0.7	0.3
Decision Memo for Infrared Therapy Devices (CAG-00291N)	158	-0.3	(-0.8, 0)	159	0	(-1, 1)	0.5	0.2

Decision Memo for Intestinal and Multivisceral Transplantation (CAG-00036N)	23	0.9	(-0.6, 4)	23	-3	(-5, -2)	0.0	0.0
Decision Memo for Leadless Pacemakers (CAG-00448N)	31	-0.1	(-1, 2)	31	-4	(-4, -4)	0.0	0.0
Decision Memo for Lumbar Artificial Disc Replacement (CAG- 00292N)	36	1.5	(-0.3, 3.9)	37	-4	(-5, -4)	0.0	0.0
Decision Memo for Lumbar Artificial Disc Replacement (LADR) (CAG-00292R)	28	0.3	(-0.8, 1.2)	28	-5	(-6, -3)	0.1	0.0
Decision Memo for Magnetic Resonance Angiography of the Abdomen and Pelvis (CAG-00142N)	20	-0.2	(-0.9, 0.3)	20	-2	(-3, -1.5)	0.2	0.0
Decision Memo for Magnetic Resonance Imaging (MRI) (CAG- 00399R)	32	0.3	(-1, 1.3)	32	-1	(-2, 3)	0.2	0.1
Decision Memo for Magnetic Resonance Imaging (MRI) (CAG- 00399R2)	27	1.4	(-0.5, 4.7)	27	-2	(-3, -1)	0.4	0.1
Decision Memo for Magnetic Resonance Imaging (MRI) (CAG- 00399R4)	27	-0.1	(-1, 0.8)	27	-4	(-5, -2)	0.5	0.4
Decision Memo for Microvolt T-wave Alternans (CAG-00293N)	20	0.9	(0.1, 2.4)	20	-3	(-4, -2)	0.3	0.0
Decision Memo for Microvolt T-wave Alternans (CAG-00293R)	23	0.1	(-0.3, 0.7)	24	-3	(-4.5, -1)	0.2	0.1
Decision Memo for Microvolt T-wave Alternans (CAG-00293R2)	40	-0.7	(-1.2, 0.5)	41	-1	(-2, 0)	0.4	0.5
Decision Memo for Neuromuscular Electrical Stimulation (NMES) for Spinal Cord Injury (CAG-00153R)	25	-0.4	(-1, -0.2)	25	-2	(-3, -1)	0.0	0.0
Decision Memo for Next Generation Sequencing (NGS) for Medicare Beneficiaries with Advanced Cancer (CAG-00450R)	27	-0.8	(-1.1, 0.1)	27	-3	(-3, -2)	0.4	0.1
Decision Memo for Ocular Photodynamic Therapy (OPT) with Verteporfin for Macular Degeneration (CAG-00066R4)	34	-0.6	(-1, 0.4)	34	0	(-1, 1)	0.0	0.1

Decision Memo for Percutaneous Image-guided Lumbar Decompression for Lumbar Spinal Stenosis (CAG-00433N)	24	-1.6	(-1.9, -0.9)	24	-4	(-6, -3)	0.3	0.4
Decision Memo for Percutaneous Image-guided Lumbar Decompression for Lumbar Spinal Stenosis (CAG-00433R)	40	-1.1	(-1.6, -0.2)	40	-3	(-4, -1.5)	0.2	0.3
Decision Memo for Percutaneous Left Atrial Appendage (LAA) Closure Therapy (CAG-00445N)	101	2.6	(1.8, 4.8)	102	-1	(-2, 0)	0.5	0.3
Decision Memo for Percutaneous Transluminal Angioplasty (PTA) and Stenting of the Renal Arteries (CAG-00085R4)	20	0	(-1.7, 7.7)	20	-1	(-3, 1)	0.2	0.1
Decision Memo for Percutaneous Transluminal Angioplasty (PTA) of the Carotid Artery Concurrent with Stenting (CAG-00085N)	44	2.8	(1.8, 4.5)	45	-3	(-3, -1)	0.4	0.1
Decision Memo for Positron Emission Tomography (FDG) (CAG- 00065N)	40	1	(0.2, 2.1)	40	-3	(-3, -2)	0.1	0.0
Decision Memo for Positron Emission Tomography (FDG) for Infection and Inflammation (CAG-00382N)	26	1.4	(0.7, 2.3)	26	-3	(-4, 0)	0.0	0.0
Decision Memo for Positron Emission Tomography (FDG) for Solid Tumors (CAG-00181R)	34	-0.4	(-0.8, 0.2)	34	-3	(-3.5, -2)	0.0	0.0
Decision Memo for Positron Emission Tomography (FDG) for Solid Tumors (CAG-00181R4)	53	-0.4	(-0.7, -0.1)	53	-3	(-4, -2)	0.6	0.1
Decision Memo for Positron Emission Tomography (N-13 Ammonia) for Myocardial Perfusion (CAG-00165N)	39	-0.1	(-1.1, 0.6)	39	2	(0, 5)	0.9	0.5
Decision Memo for Positron Emission Tomography for Initial Treatment Strategy in Solid Tumors and Myeloma (CAG-00181R3)	36	-0.1	(-0.8, 0.2)	36	-3	(-3, -2)	0.1	0.0
Decision Memo for Screening for Colorectal Cancer - Stool DNA Testing (CAG-00440N)	22	2.5	(0.4, 11.1)	22	-1	(-4, 3)	1.2	0.4
Decision Memo for Screening for Lung Cancer with Low Dose Computed Tomography (LDCT) (CAG-00439N)	38	2.4	(0.4, 6)	38	-2	(-2.5, 0)	1.1	0.5

Decision Memo for Screening for the Human Immunodeficiency Virus (HIV) Infection (CAG-00409N)	32	1.5	(0, 3)	32	-4	(-4, -3)	0.5	0.9
Decision Memo for Serum Iron Studies (Addition of Restless Leg Syndrome as a Covered Indication) (CAG-00263R)	60	1.2	(0.5, 2.3)	65	0	(-2, 0)	0.1	0.4
Decision Memo for Sleep Testing for Obstructive Sleep Apnea (OSA) (CAG-00405N)	59	1	(0.6, 1.8)	59	0	(-2, 1)	0.5	0.3
Decision Memo for Stem Cell Transplantation (Multiple Myeloma, Myelofibrosis, and Sickle Cell Disease) (CAG-00444R)	37	1.8	(1.5, 2.9)	38	1.5	(-1, 3)	0.8	1.2
Decision Memo for Supervised Exercise Therapy (SET) for Symptomatic Peripheral Artery Disease (PAD) (CAG-00449N)	48	-0.4	(-1.2, 0.3)	48	0.5	(-1, 2)	0.4	0.8
Decision Memo for Surgery for Diabetes (CAG-00397N)	47	1.5	(0.3, 3.3)	47	-1	(-2, 0)	0.2	0.1
Decision Memo for Thermal Intradiscal Procedures (CAG-00387N)	140	-1.3	(-1.5, -1)	141	-2	(-2, -1)	0.5	0.0
Decision Memo for Transcatheter Aortic Valve Replacement (TAVR) (CAG-00430N)	33	2.7	(0.2, 6.2)	33	-4	(-4, -3)	0.1	0.1
Decision Memo for Transcatheter Aortic Valve Replacement (TAVR) (CAG-00430R)	141	1.6	(1.2, 2.8)	141	-2	(-2, -1)	1.0	0.4
Decision Memo for Transcatheter Mitral Valve Repair (TMVR) (CAG- 00438N)	21	1.5	(-0.5, 4.6)	21	-4	(-4, -3)	0.1	0.0
Decision Memo for Transcutaneous Electrical Nerve Stimulation for Chronic Low Back Pain (CAG-00429N)	48	-0.3	(-0.9, 0.1)	49	1	(-2, 4)	0.1	0.0
Decision Memo for Vagus Nerve Stimulation (VNS) for Treatment Resistant Depression (TRD) (CAG-00313R2)	24	1.8	(1, 4.5)	24	-1	(-2.5, 2.5)	0.9	0.3
Decision Memo for Vagus Nerve Stimulation for Treatment of Resistant Depression (TRD) (CAG-00313R)	37	1.8	(-0.1, 2.4)	38	-4	(-5, -2)	0.8	0.1

Decision Memo for Ventricular Assist Devices for Bridge-to- Transplant and Destination Therapy (CAG-00432R)	43	4.7	(0.4, 8.2)	43	-3	(-4, -2)	0.6	0.2
Proposed Decision Memo for Erythropoiesis Stimulating Agents (ESAs) for Treatment of Anemia in Adults with CKD Including Patients on Dialysis and Patients not on Dialysis (CAG-00413N)	577	-1.1	(-1.2, -0.9)	597	5	(4, 6)	0.6	0.2

Notes. This table shows the median ΔRCR and $\Delta Year$ by memo (N = 68). For each memo, the number of memo articles with non-provisional RCR (# Memo Articles w/ RCRs) and publication year (# Memo Articles w/ Years) data are recorded. The same values are reported for the comparison set. ΔRCR and $\Delta Year$ values for each memo are reported along with the 95% confidence interval (bracketed numbers) as described in the Methods. Only those memos with at least twenty memo articles are shown. Kullback-Leibler Divergence ("KLD") columns are calculated as described in the Supplemental Methods with numbers closer to zero indicating a smaller divergence from a uniform distribution (e.g., all funders/research organizations support equal shares of articles cited in memo). KLD is reported for funders (KLD_F) and research organizations (KLD_{RO}).

Supplemental Methods

Assigning award years for funding associated with memo articles

Associating project (i.e., award) data with articles using PubMed and RePORTER is non-trivial. Often an abbreviation of the project number (e.g., the core project number) is reported, which can be associated with multiple project years. For instance, a scientist may receive a 3-year award and, at the conclusion of the first award, apply for a second 3-year award based on the results produced by the first award. If the scientist is successful, NIH appends a new suffix (e.g., "-02") to the core project number (e.g., "R01CA031770") assigned to the first project. Often, scientists will only report the core project number in a publication, which creates ambiguity when assigning a specific year to the cited funding. Similarly, RePORTER uses publication data reported in award applications to link articles to funding (NIH, 2024).

Unfortunately, inclusion of an article in an award application indicates the research was done prior to its being cited in that application. In both cases the year can be imputed by using award history data available in RePORTER.

I imputed the year for each award from an NIH Institute or Center (IC) that had records available in RePORTER (N = 2,444)[iv]. First, all projects (i.e., full project numbers) associated with a core project number were retrieved from RePORTER and linked to

[iv] This number is greater than the total number of unique projects funded by NIH ICs (N = 1,996) because the unique count only considers core project numbers (e.g., R01CA031770). For this analysis, full project numbers (e.g., R01CA031770-01) are imputed using the article publication date. Therefore, some core project numbers may be repeated (e.g., R01CA031770-01, R01CA031770-03, etc.) since they are cited by multiple publications from different years.

the memo article that cited their core project number. Each project was then compared to the citing article's publication year (article publication year - project year) and the project with the difference closest to one was assigned as the award year used in subsequent calculations described in the Methods section of the main paper. I used this procedure because I assumed that more recent awards were more relevant to an article that cited the associated core project number but that awards made in the same year as the publication or after it were less relevant (though not excluded). For awards that could not be linked to a record in RePORTER, I assumed that the award was issued the year prior to the publication of the article citing it.

Measuring concentration in funders and research organizations by memo

Data on the funders and organizations involved in the memo research ecosystem can also be used to measure whether few or many funders/research organizations support the articles cited by CMS memos. I use the Kullback-Leibler Divergence (KLD) to measure the divergence of the observed distribution of funders/research organizations from a uniform distribution (e.g., where all proportions are equal) using equation S1:

$$(S1) \qquad KLD_x = \sum_{i}^{N} p_i \ln(N * p_i)$$

where x is the individual memo, i is the funder or research organization, N is the total number of funders or research organizations supporting the memos cited in x, and p_i is the proportion of articles supported by funder or research organization i ($\sum_i^N p_i = 1$). KLD_x values are approximations because proportions were calculated using the number

articles with funder/research organization information in place of the total number of articles cited in a memo (many articles could not be linked to funder/research organization data). For articles citing multiple funding/research organizations, the count for the article is divided into equal fractions for each entity (e.g., each funder would receive 0.25 counts for a memo article supported by four different funders). The distribution of KLD for funders can be compared to that of research organizations using a paired Wilcoxon signed rank test.

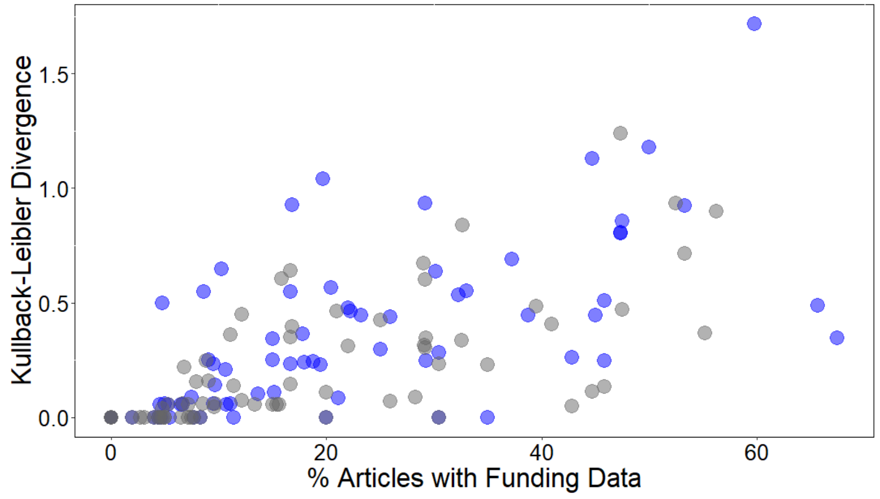

Figure S1. Kullback-Leibler Divergence (KLD) by memo. Graph plotting the KLD of funders (blue) and research organizations (gray) against the number of articles in each memo that contained data on funder and/or research organization. Only memos linked to at least twenty research articles are shown.

Figure S1 shows that memos take on a wide range of KLD values. The KLD distribution for funders is statistically different from that of research organizations (pseudo-median of the difference = 0.14; p-value = $2E^{-6}$), indicating that the distribution of support for memo articles among funders is more skewed than it is for research organizations. In

other words, support for articles is distributed less equally among funders (i.e., some funders support a larger share of articles) than among research organizations.

References

NIH. (2024). *Research Portfolio Online Reporting Tools Expenditures and Results (RePORTER)* https://reporter.nih.gov/publications
